# Shifting equilibria in a warming boreal forest

**DOI:** 10.1073/pnas.2424669122

**Published:** 2025-01-13

**Authors:** Troy S. Magney, Zoe A. Pierrat

**Affiliations:** ^a^Department of Plant Sciences, University of California, Davis, CA 95616; ^b^Jet Propulsion Laboratory, California Institute of Technology, Pasadena, CA 91011

The boreal forest is shifting, driven by warming temperatures, altered precipitation patterns, and intensifying disturbances (1–3). Shifts in tree cover dynamics can be key indicators of broader boreal biome transformations (4), evidenced by patterns of tree growth, dieback, mortality, and changes in vegetation composition (5–7). Satellite and ground data reveal signs of northward range expansion, increased tree density in the core and declines at southern margins in recent decades (8–10). The trajectory of these changes will depend on future climate scenarios, with implications for the carbon cycle, biodiversity, and ecosystem services (2). In PNAS, Rotbarth et al. (11) examine two decades of boreal tree cover changes from satellite imagery and project how rising temperatures could shape their future.

Rotbarth et al. suggest that the current bimodal distribution of tree cover—dense forests (>60% cover) and sparse woodlands (<15% cover)—could transition to a more uniform “open forest” state with 30 to 50% cover by 2100. This shift results from thinning tree cover in southern forests and northern areas densifying. While this redistribution could enhance carbon storage in some regions, it underscores the instability of intermediate states, which are particularly vulnerable to abrupt changes. Notably, detecting these transitions across large scales is dependent on the spatiotemporal resolution of available data.

The best available long-term satellite records from Landsat (30 m pixels) and MODIS (250 to 500 m pixels) struggle to capture the patchy, heterogeneous nature of boreal vegetation, particularly in transitional or open forests. Understory vegetation, which appears green to satellites, can obscure true tree cover estimates. Snow, soil background, high cloud cover, and low solar angles in high-latitude regions further complicate satellite interpretation of forest structure and function (12). As such, misclassification errors are common, particularly in regions where vegetation falls below the resolution threshold of remote sensing imagery (7, 13). Further complicating this, the short temporal window of satellite observations—typically 20 to 40 y—often entails a space-for-time substitution which limits our ability to differentiate gradual change from abrupt transitions. Long-term paleoecological datasets suggest that over the past 8,000 y, boreal forests have responded to climatic changes gradually due to stabilizing feedbacks (14). However, long-term climatic changes have also been more gradual than those in the modern era. Therefore, by combining insights from long-term records, controlled experiments, and satellite observations, we can better understand how both stabilizing and tipping point feedbacks will ultimately impact a shifting equilibrium in the boreal.

## Climate Feedbacks and Ecological Consequences

**Fig. 1.**
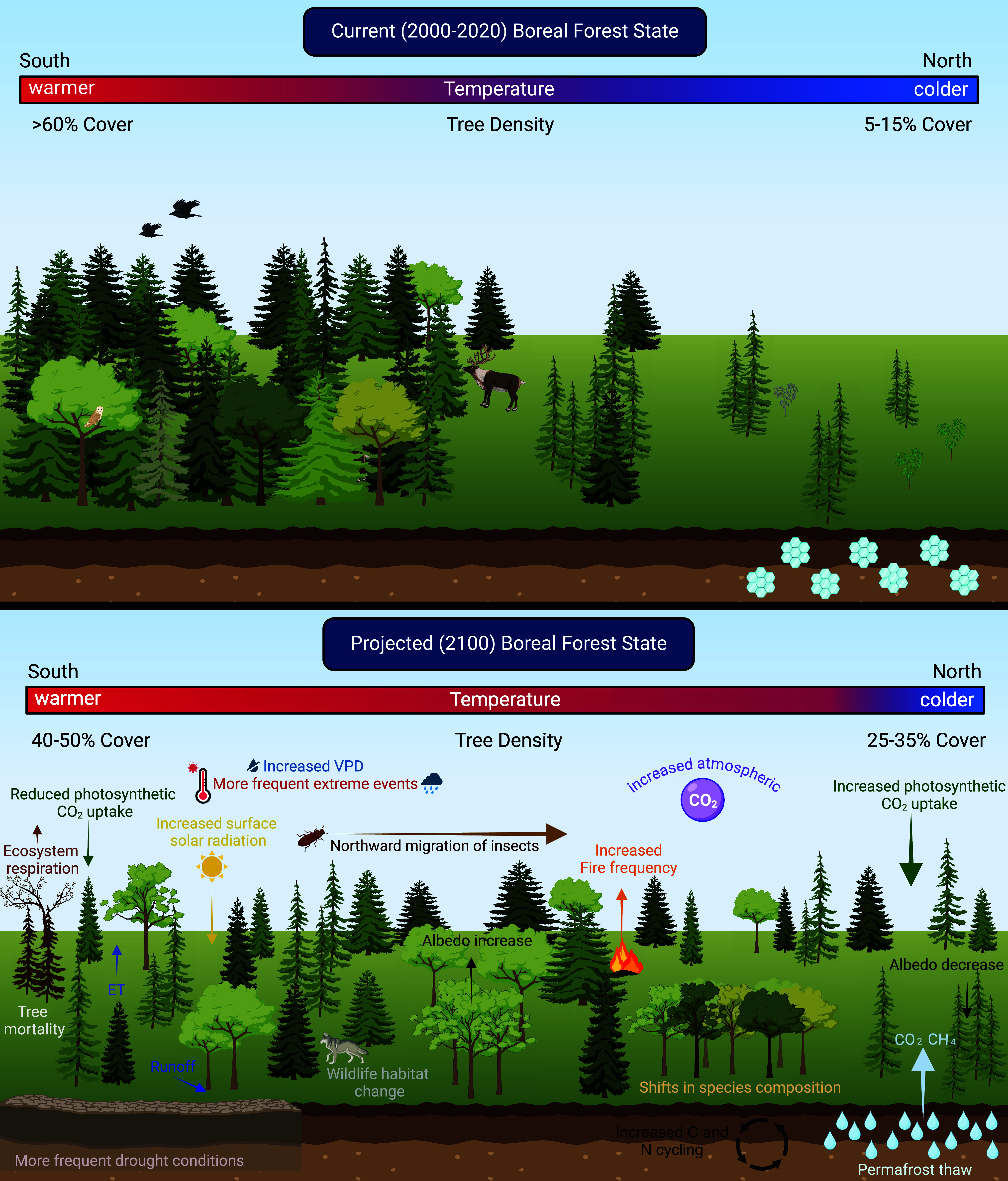
Simplified conceptual diagram of current tree cover distribution (*Top*) and potential feedbacks associated with a projected open forest state in the year 2100 (*Bottom*). Image created using Biorender.com.

The transition to an open forest state is closely tied to feedback processes such as changes in biomass and species composition, disturbance (wildfire, insects, wind), albedo, water availability, and permafrost dynamics (Fig. 1). Addressing these dynamics requires careful consideration of tradeoffs between feedback in a spatially explicit manner that accounts for varying topographies, climates, forest management practices, stand ages, and species compositions. These recent projections (11) raise critical questions: If the boreal forest is indeed headed for an open state, *how will tree cover dynamics influence—and be influenced by—climate feedbacks? And how will this impact ecosystem services and the carbon budget of the boreal forest?*

Rotbarth et al. (11) estimate that the transition could result in a net carbon gain of 17.7 Gt by 2,100—a 11.4% increase compared to current levels. Dense, high-biomass forests are expected to lose biomass, while sparse, low-biomass forests could gain it (11). However, the authors acknowledge that this projection does not consider feedbacks associated with an open forest state, which would likely increase carbon losses (Fig. 1). The overall carbon sink capacity of boreal forests may be reduced due to several key factors: widespread tree mortality, the eventual leveling-off of biomass gains as forests reach their growth limits, shorter forest heights reducing biomass density, and wildfire and permafrost thaw releasing vast amounts of stored carbon (15).

Altered fire regimes are among the most critical feedbacks. Warmer temperatures and drier conditions are increasing the frequency and severity of wildfires, disrupting the traditional fire-return intervals that have historically shaped boreal forests (5). Open forests, with sufficient fuel and favorable microclimatic conditions, are especially vulnerable to fire (16). They may be more susceptible to short-interval fires—occurring less than 40 y apart—preventing full recovery, reducing biomass, and shifting species composition (evergreen to deciduous) (5).

Changes in forest density and composition will have a significant impact on surface albedo (17). Initially, burnt areas will decrease albedo (increasing surface warming), but postfire recovery often favors deciduous species, which can increase surface reflectivity (albedo) and provide temporary cooling effects. However, these effects are spatially and temporally variable and are often reversed as evergreen conifers reestablish (5). In northern regions, where forests are densifying, reduced albedo may exacerbate warming, potentially offsetting the benefits of increased carbon storage.

Future water availability is also already being altered through complex and region-specific patterns, with implications for future forest dynamics (18, 19). Higher evapotranspiration rates and reduced summer precipitation are straining soil moisture, especially in southern regions (18). Water availability could continue to decline if forests in southern regions enter a more open state by increasing runoff and allowing more solar radiation to reach the soil and snowpack, increasing evaporative losses. Future precipitation patterns are anticipated to shift toward fewer but more intense rainfall events, interspersed with longer and more severe droughts (18). These intensified rainfall events could enhance temporary infiltration but prolonged dry spells exacerbate plant stress due to elevated vapor pressure deficits and increased surface solar radiation.

In the mid-southern boreal, future climate warming, increased evapotranspiration, and reduced soil moisture are likely to drive stronger stomatal constraints on photosynthesis (20). This could diminish or negate the potential carbon gains from increased photosynthetic capacity and anticipated growth (21). Increased precipitation in winter and less in summer exacerbates water stress during critical growing periods (3). Earlier spring photosynthesis due to warming could lead to increased carbon uptake (21), particularly during snow-on periods (22), but lead to earlier depletion of water resources, and ultimately mortality (3, 23). Conversely, warmer temperatures could enhance productivity in colder, wetter regions, offsetting negative impacts of increased forest disturbance and thinning of the southern boreal (1). Across the boreal, the net impact of these changes in tandem with increases in atmospheric carbon dioxide impacting forest water-use efficiency are still unclear, as direct experiments testing the influence of climate warming and tree density under diverse soil moisture conditions are scarce, but critical (3).

Taken together, the boreal forest’s transition to an open state has potentially large implications for climate regulation, biodiversity, and ecosystem services.

A change in tree cover across the boreal may alter permafrost dynamics. Permafrost thawing, which increases availability of soil nutrients, alters drainage patterns, and creates localized waterlogged or dry conditions, leading to varied vegetation responses (24)—making it difficult to account for in model projections. Such heterogeneity challenges the uniformity of the predicted open forest state and suggests that regional variability in feedback mechanisms could lead to diverse forest trajectories. Nevertheless, widespread permafrost thaw will continue regardless of changing forest density and will remain a major contributor to greenhouse gas amplification (14).

The increased frequency of extreme events, such as heatwaves and droughts, further intensifies water stress, reducing forest resilience to disturbance from pathogens and insects (23). Documented expansions in insect ranges and outbreak severity across Europe and North America (25) are linked to temperature-mediated changes in phenology and interactions between host trees and herbivorous insects (24). Increases in temperature and tree density northward could further expand their range, reducing forest carbon uptake via photosynthesis.

Reduced forest density and shifting species distributions disrupt habitats and predator–prey dynamics. For example, boreal woodland caribou are losing critical habitat as forests thin, while wolves and deer expand their ranges (26). The loss of old-growth forests, replaced by younger, managed stands, further threatens biodiversity. These mature forests provide structural legacies critical for many birds, native insects, and small mammals, and their decline represents a significant reduction in ecosystem resilience (26).

Taken together, the boreal forest’s transition to an open state has potentially large implications for climate regulation, biodiversity, and ecosystem services. This awareness creates opportunities to mitigate the risks associated with this transition. To understand how, developing future model projections that can account for the interlocking feedbacks of a forest in transition are critical. For example, *how might a shift to an open state accelerate or delay feedbacks? Will carbon increases in northern tree growth offset decreases in the south? How will albedo, disturbance regimes (wildfire, insects, pathogens, wind), water availability (including snowpack), and permafrost be impacted?* Answering questions like this will require multidiscipline and multiscale syntheses (12); but ultimately, to slow the potential impacts associated with an open forest state, we must curb greenhouse gas emissions and adopt adaptive management strategies that enhance forest resilience, sustain human livelihoods and biodiversity, and preserve the essential role of the boreal region in climate regulation.
